# SAPK2 contributes to rice yield by modulating nitrogen metabolic processes under reproductive stage drought stress

**DOI:** 10.1186/s12284-020-00395-3

**Published:** 2020-06-08

**Authors:** Dengji Lou, Zhen Chen, Diqiu Yu, Xiaoyan Yang

**Affiliations:** 1 School of Chemical, Biological and Environmental Sciences, Yuxi Normal University, Yuxi, 653100 China; 2 Faculty of Life Science and Technology, Kunming University of Science and Technology, Kunming, 650500 Yunnan China; 3 State Key Laboratory for Conservation and Utilization of Bio-Resources in Yunnan, Yunnan University, Kunming, 650091 China

**Keywords:** Rice, *SAPK2*, Grain yield, Grain size, NO_3_^−^ influx, Drought stress, Nutrient-deprived

## Abstract

**Background:**

The sucrose non-fermenting 1-related kinases 2 (SnRK2s) play important roles in osmotic stress responses in *A. thaliana* and rice (*Oryza sativa* L.). Osmotic stress/ABA–activated protein kinase 2 (SAPK2) is a member of SnRK2s subclass II in rice, but its function in rice yield under drought stress is unclear.

**Results:**

Compared with wild-type (Oryza.Sativa L.spp.japonica, WT) plants, the *sapk2* rice mutant lines were shorter and produced fewer grains per panicle, smaller grains and lower grain yield under reproductive stage drought stress (RDS). Subsequent analysis suggested that SAPK2 considerably influences the nitrogen, phosphorus, and potassium contents of rice grains. The examination of rice seedling growth and development under nutrient-deprived conditions (−N, −K, and − P) proved that SAPK2 can significantly affect rice seedling growth and root development in hydroponic cultures lacking N and K. Moreover, the NO_3_^−^ influx rate and nitrate concentration analysis indicated that SAPK2 promotes nitrate uptake and assimilation by regulating nitrate-related transporters.

**Conclusion:**

These results suggest that *SAPK2* could enhance grain production by regulating nitrogen utilization efficiency under RDS. Our work provided insights to breeding drought tolerant rice with high nutrient uptake.

## Background

The sustainability of crop production has been challenged by climate changes, an insufficient freshwater supply, and an increasing global population (Comas et al. 2013; Gray and Brady 2016). Additionally, drought stress, which is important abiotic factor limiting crop productivity worldwide, is responsible for extensive crop losses and will likely worsen in the near future. Thus, there is increasing international interest in developing drought-tolerant crops. In rice, plant architecture and grain yield are affected by environmental conditions and genetics. Enhancing the drought tolerance of rice is challenging because of the complexity of this trait and the incomplete characterization of the physiological and molecular mechanisms associated with drought responses. Accordingly, considerable work is required to address the adverse effects of drought stress and ensure the food security of future generations.

Drought stress can affect plants at all growth stages, and the extent of the changes to productivity depend on the plant species and its genotype, age, and size as well as the duration and severity of the stress (Gall et al. 2015). Plant growth is highly sensitive to water deficit, largely because of the resulting inhibition of cell elongation. Water-stressed plants are shorter than normal and have a smaller leaf area, which decreases the amount of photo synthetically active radiation absorbed by leaves, the photosynthetic rate, and ultimately yield. Water deficit induces stomata closure, which leads to limited CO_2_ uptake by leaves and decreased net photosynthesis (Yang et al. 2017). It also affects carbohydrates, the ATP content, respiration, and abscisic acid (ABA). Moreover, it leads to the excessive accumulation of reactive oxygen species, resulting in oxidative stress that seriously damages cellular functions (Deeba et al. 2012). In addition to its direct impact on plants, drought stress also has various indirect effects on crop growth and yield. Nutrients, especially macronutrients, are very important for plant growth and yield, and their uptake is restricted under drought stress conditions (Aroca 2012). The uptake of nitrogen (N) and potassium (K) reportedly decreases in cotton plants exposed to drought stress (McWilliams 2003). The concentration of growth-retarding substances usually increases under stress conditions to adjust plant water levels for various processes (Farooq et al. 2009).

Plant responses to drought stress include stress signal perception, signal transduction and amplification, and adaptations at the morphological, physiological, and molecular levels. Phytohormones are the key mediators of plant responses to drought stress (Sah et al. 2016). Water deficit also alters the endogenous synthesis of various phytohormones, including jasmonic acid (JA), ABA, salicylic acid (SA), ethylene (ET), auxin, gibberellins (GAs), and cytokinins (CKs). ABA is the major stress-responsive hormone produced after the drought signal is perceived by plants. Osmotic stress promotes ABA synthesis, which activates gene expression and adaptive physiological changes (Yamaguchi-Shinozaki and Shinozaki 2006). Additionally, ABA remains the best-studied hormone for plant stress responses, and the interaction between ABA and other classical stress-related hormones enables plants to rapidly respond and properly adapt to drought stress (Brodribb and McAdam 2017). The ABA-dependent signaling pathways are critical for the expression of genes responsive to various stresses, especially osmotic stress. SnRK2 family members are protein kinases which promote ABA responses (Hrabak et al. 2003).

To date, 10 plant-specific SnRK2s have been identified in *Arabidopsis thaliana* (SnRK2.1–2.10) and rice (SAPK1–10) (Kobayashi et al. 2004). In *A. thaliana*, SnRK2.2, SnRK2.3, and SnRK2.6 are involved in ABA responses (Fujii and Zhu 2009). For example, upon hyperosmotic stress, ABA accumulates and binds to the PYR/PYL/RCAR-type ABA receptors, which subsequently inhibit PP2C activity, resulting in the release of SnRK2s from inhibition, and then the activated SnRK2s phosphorylate downstream effectors to mediate stress responses (Fujii et al. 2009; Ma et al. 2009; Park et al. 2009; Santiago et al. 2009; Umezawa et al. 2009). Additionally, in rice, members of SnRK2 subclass III (SAPK8–10), as well as SAPK2, help regulate ABA-dependent gene expression via the ABA signal transduction pathway mediated by OsPYL/RCARs and PP2Cs (Kim et al. 2012).

A recent study confirmed that the overexpression of *SAPK9* may significantly enhance drought tolerance, while also increasing the grain yield under drought condition (Dey et al. 2016). In previous research, we found that the *sapk2* mutants exhibited an ABA-insensitive phenotype during the germination and post-germination stages and sensitive phenotype to drought stress, with lower survival rates than the wide-type plants. The mutants also exhibited greater water loss, lower proline and soluble sugar contents, higher proportions of fully open stomata, higher ROS levels, and lower antioxidant enzyme activities (Lou et al. 2017). These results indicate that SAPK2 may be useful for improving crop yields under drought conditions. However, the effect of SAPK2 on the productivity under drought stress remains unclear.

In this study, we examined knock-out mutant lines (*sapk2*; *sapk2–1* and *sapk2–7*), which we previously developed with the CRISPR/Cas9 system, and *SAPK2*-overexpressing lines (*OE*; *OES2–1* and *OES2–2*). We found that the *sapk2* mutant showed lower grains yield and lower nitrogen, phosphorus, and potassium contents of rice grains than the WT RDS. Moreover, the *sapk2* mutant exhibited weaker seedling growth and root development in hydroponic cultures lacking N and K. The NO_3_^−^ influx rate and nitrate concentration analysis indicated that SAPK2 promotes nitrate uptake and assimilation by regulating nitrate-related transporters. Our work provided insights to breeding drought tolerant rice with high nutrient uptake.

## Methods

### Generation of Transgenic Rice Lines

We employed knock-out mutant lines (*sapk2*, *sapk2–1* and *sapk2–7*) which we built previously by the CRISPR/Cas9 system and over-expression lines (*OE*, *OES2–1* and *OES2–2*) (Lou et al. 2018). Concisely, the third coding exons of *SAPK2* were selected for guide RNA design. Double-strand DNA generated by annealing the oligo pairs, and then was cloned into the pYLCRISPR/Cas9Pubi-H vector. For mutation detection, genomic DNA extracted from mutant seedlings (all plant) were used for PCR. In T_0_ generation, we collected 20 hygromycin-resistant plants for each gene. Based on mutation detection results, we identified two independent homozygous mutant lines in the T_1_ generation, which we named *sapk2–1, sapk2–7*. To generate *SAPK2* overexpression transgenic plants, the full-length cDNA of *SAPK2* was cloned into the p1301 vector in the sense orientation behind the CaMV 35S promoter. Rice (*Oryza sativa* L. japonica.) was used for transformation. The primers used in this study were listed in the Additional file 1: Table S1.

### Plant Cultivation and Agronomic Traits Analysis

For basic agronomic traits analysis, rice plants were grown in the paddy field from March to August at the rice experimental station of Xishuangbanna Tropical Botanical Garden. Ten plants at a spacing of 16.5 cm × 26.5 cm were planted in a row and 5 rows of each line were planted. At reproductive stage, 20 plants of each line were randomly chosen to detect agronomic traits. The grain number per panicle was measured as the total number of grains per plant divided by the number of panicles per plant. The 1000-grain weight was calculated as the weight of the total grains per plant and divided by the grain number, then converted to 1000-grain weight. Grain yield per plant was measured as the weight of total grains per plant.

### Reproductive-Stage Drought Stress and Nutrient-Deprived Treatments

For reproductive-stage drought stress, transplanted experiments were maintained as described by Sandhu et al. (2016). The drought stress was initiated at 32 days after transplanting and continued for 30 days. After the inception of the stress, the soil water potential was measured using tensiometers (30 cm depth). The plots in the reproductive stage drought stress treatments were rewatered when the soil water potential dropped to − 50 to − 70 kPa (tensiometer). The decline in water table depth was measured on a daily basis with a meter scale inserted into a 1.1-m polyvinyl chloride pipe in the experimental fields at regular intervals in all RDS treatments. The pipes were placed at 1.0-m depth with 10 cm of pipe remaining above the soil surface. The plots were rewatered when water table level reached 100 cm below the soil surface and most lines were wilted and exhibited severe leaf drying.

To analyze *SAPK2* function in seedling growth and development under different nutrient-deprived conditions, seedlings at 7 DAG were cultured in basic nutrient solution (pH = 5.8) for a week. Then seedlings at 14 DAG were transferred to nutrient-deprived solutions. Each nutrient solution was renewed every 3 days. Daytime conditions in the greenhouse were 32 °C, with light from a sodium lamp (400 W) for 14 h; night-time conditions were 25 °C, and dark for 10 h. At 34 DAG, root length, shoot length, root number and dry weight of each lines were measured.

### Measurement of Nitrate, Phosphorus, Potassium and Total Nitrogen Content and Nitrate Influx

For free NO_3_^−^ analysis, 0.1 g plant materials from different genotypes were homogenized by grinding in cold extraction buffer [50 mM Tris–HCl (pH 7.0), 10 mM imidazole, and 0.5% (w/v) b-mercaptoethanol]; the homogenates were then centrifuged at 12, 000 g for 20 min at 4 °C and the supernatant was collected. Free NO_3_^−^ in the supernatant was determined by using the Griess method (Walther et al. 1999), the absorbance at 540 nm was determined and NO_3_ contents were calculated from the standard curve of KNO_3_.

NO_3_^−^ influx was calculated as the difference in NO_3_^−^ content between the plants under control conditions and drought stress conditions in an hour.

For total nitrogen, phosphorus and potassium content analysis, 50 seeds from different genotypes were homogenized by grinding in Liquid nitrogen. Then 0.1 g seed materials were measured using Agilent 7700e ICP-MS.

At least three independent biological experiments were conducted. One representative result was displayed here.

### RNA Extraction and qRT-PCR Analysis

To detect the transcript level of *SAPK2* under different nutrient-deprived conditions, 14 DAG seedlings were transferred to different nutrient-deprived solutions for 24 h. To detect the transcript level of target genes under drought stress, seedlings were transferred into half-strength liquid medium supplemented with 25% PEG6000 (m/v) for 24 h. All samples were collected at the right time.

For the qRT-PCR analysis, we used the same method as described (Jiang et al. 2016). Total RNA was isolated from whole seedlings using the TriZol reagent (Invitrogen). The cDNAs were obtained by using Superscript II in accordance with manufacturer’s instructions (Invitrogen). The qRT-PCR analysis was performed using SYBR Premix Ex Taq kit (Takara).

At least three independent biological experiments were conducted (three independent samples were conducted for each experiment and three technological replications in every independent experiment). One representative result was displayed here. Gene-specific primers used in qRT-PCR analysis were listed in Additional file 1: Table S1.

### Statistical Analysis

The experiments were arranged in a completely randomized design with at least three replicates for each treatment. Excel 2010 was used for making charts. Two-tailed Student’s t tests were performed using the SPSS 10 software (IBM, Inc.). “* and **” indicate significance at *P* < 0.05 and *P* < 0.01, respectively. The data represent mean ± standard error (SE) of three independent experiments.

## Results

### Mutations to *SAPK2* Decrease Plant Height and Grain Yield

**Fig. 1:**
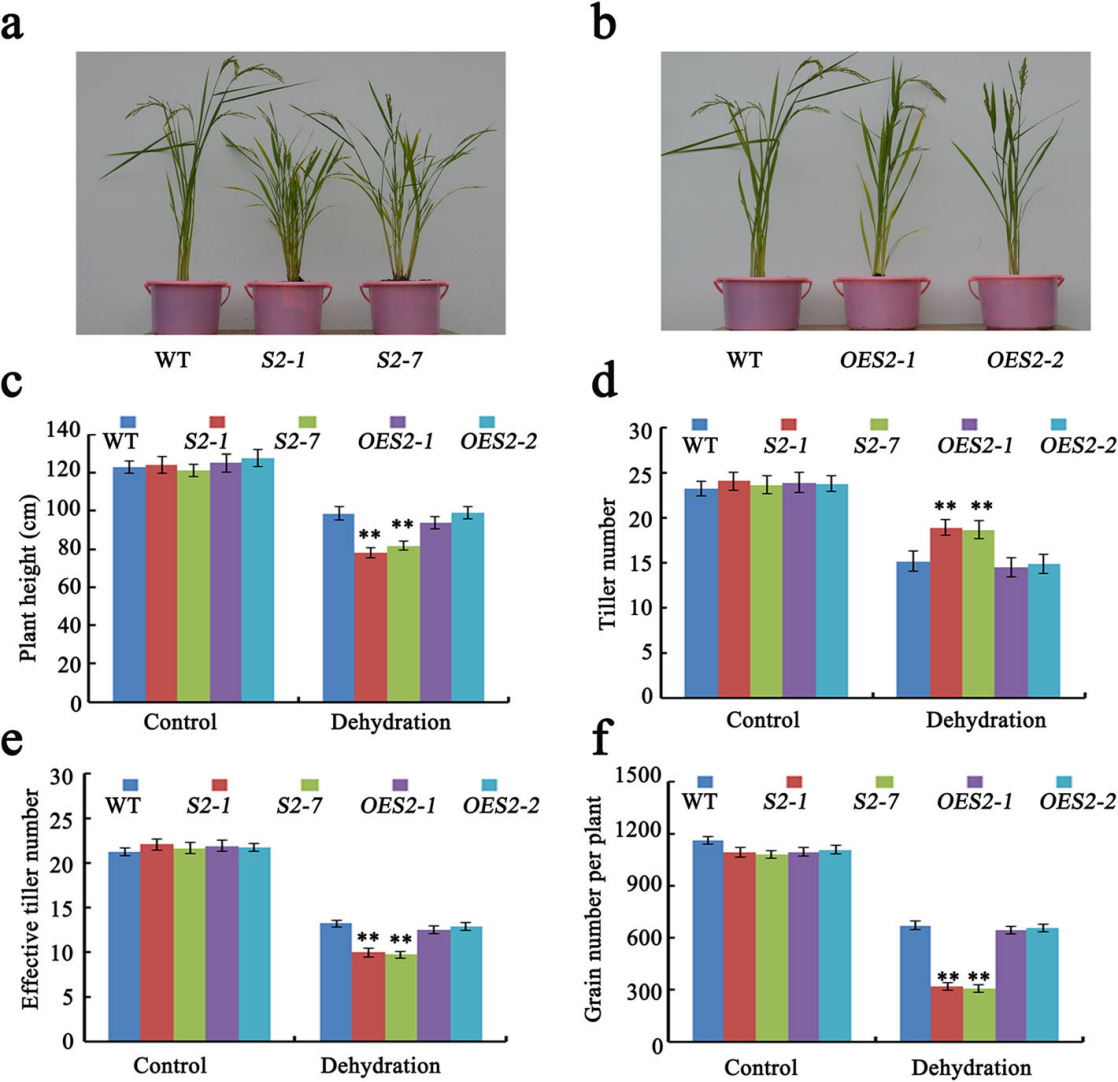
*SAPK2* has large effects on plant height and grain number under RDS for 30 days. **a** Phenotypes analysis of WT and *sapk2* mutant lines at mature stage under RDS. **b** Phenotypes analysis of WT and *OE* lines at mature stage under RDS. **c-f** Comparison of agronomic traits including plant height **(c),** tiller number**(d),** effective tiller number (**e**), grain number per plant (**f**) among WT, *sapk2* mutant lines and *OE* lines under RDS. Data in **c-f** are shown as means ± SD (*n* = 20) from three replicates. A student’s *t*-test was used to generate *P* values; “**” indicate significance at *P* < 0.01

Under drought conditions, the two *sapk2* lines were shorter than the WT control plants (Fig. 1a–c). To further investigate the SAPK2 roles influencing rice yield, we analyzed *OE* lines (*OES2–1* and *OES2–2*) and *sapk2* knock-out mutant lines (*sapk2–1* and *sapk2–7*). An analysis of plants exposed to RDS indicated that the two *sapk2* lines had substantially more tillers than the WT plants (Fig. 1a, d), whereas there were no significant differences in the two *OE* lines (Fig. 1b–d). However, although they had more tillers, the *sapk2* mutant plants produced fewer grains per plant than WT under RDS (Fig. 1f). A subsequent investigation of the regulatory effect of SAPK2 on the number of effective tillers revealed that the *sapk2* mutant lines had considerably fewer effective tillers than WT, but there were no significant differences in the *OE* lines (Fig. 1e). This result further confirmed that the *sapk2* mutant produces fewer grains than WT under RDS.

**Fig. 2:**
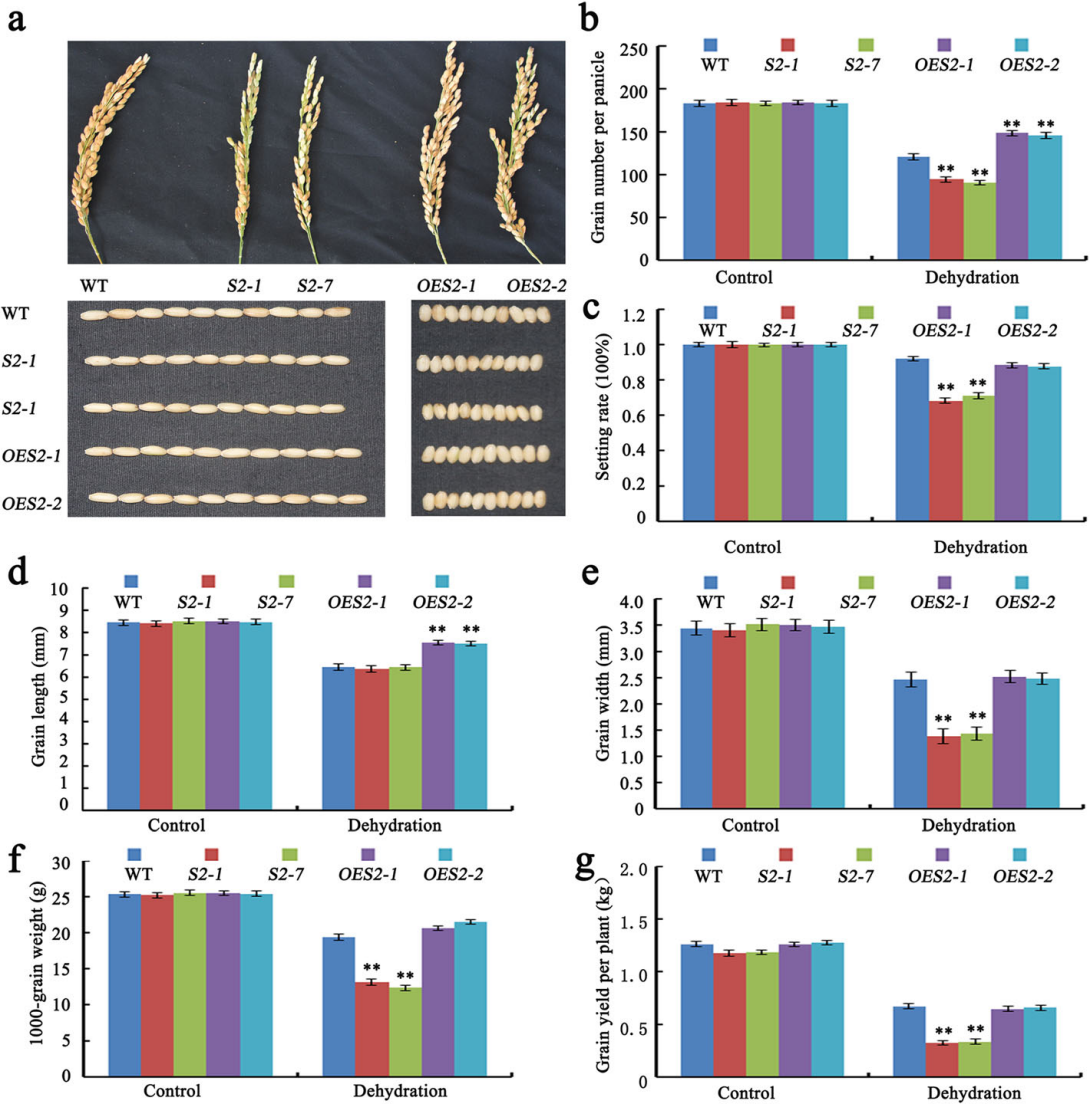
*SAPK2* has large effects on grain size and weight under RDS for 30 days. **a** Panicle and grain phenotypes of WT, *sapk2* mutant lines and *OE* lines under RDS. **b-f** Comparison of agronomic traits including grain number per panicle**(b)**, setting rate (**c**), grain length (**d**), grain width (**e**), 1000-grain weight (**f**), grain yield per plant (**g**) among WT, *sapk2* mutant lines and *OE* lines under RDS. Data in (**b-f**) are shown as means ± SD (*n* = 20) from three replicates. A student’s *t*-test was used to generate *P* values; “**” indicate significance at *P* < 0.01

The number of grains per panicle is one of the three key factors determining rice grain yield (Xing and Zhang 2010). Thus, we investigated the SAPK2 roles related to panicle and grain development by analyzing the number of grains per panicle, the seed setting rate, the grain length and width, the 1000-grain weight and the grain yield per plant in response to RDS. Under RDS, the panicles and grains of the *sapk2* mutant lines were smaller than those of WT (Fig. 2a). Additionally, the grain number per panicle of the *sapk2* mutant lines was 75% and 60.8% of that of WT and *OE* lines (Fig. 2b). The setting rate of *OE* lines did not differ from that of WT, whereas the setting rate of the *sapk2* mutant lines decreased significantly (76.9% of that of WT) (Fig. 2c). Compared with WT, the grains of *OE* lines were significantly longer, whereas there was no significant difference in the grain length of the *sapk2* mutant lines (Fig. 2d). In contrast, the grains of the *sapk2* mutant lines were significantly thinner than WT, whereas the grain width of *OE* lines was not significantly different (Fig. 2e). Moreover, the 1000-grain weight and grain yield per plant of the *sapk2* mutant lines were much lower than WT and *OE* plants (Fig. 2f, g). These results implied that SAPK2 influences panicle and grain sizes in rice under RDS.

Overall, our observations indicated that knocking out of *SAPK2* significantly decreases rice plant height and grain yield per plant under drought condition. Additionally, overexpressing *SAPK2* does not appear to enhance rice plant growth or grain production.

### Mutations to *SAPK2* Decrease Nitrate, Phosphorus, and Potassium Contents in Rice Grains under RDS

**Fig. 3:**
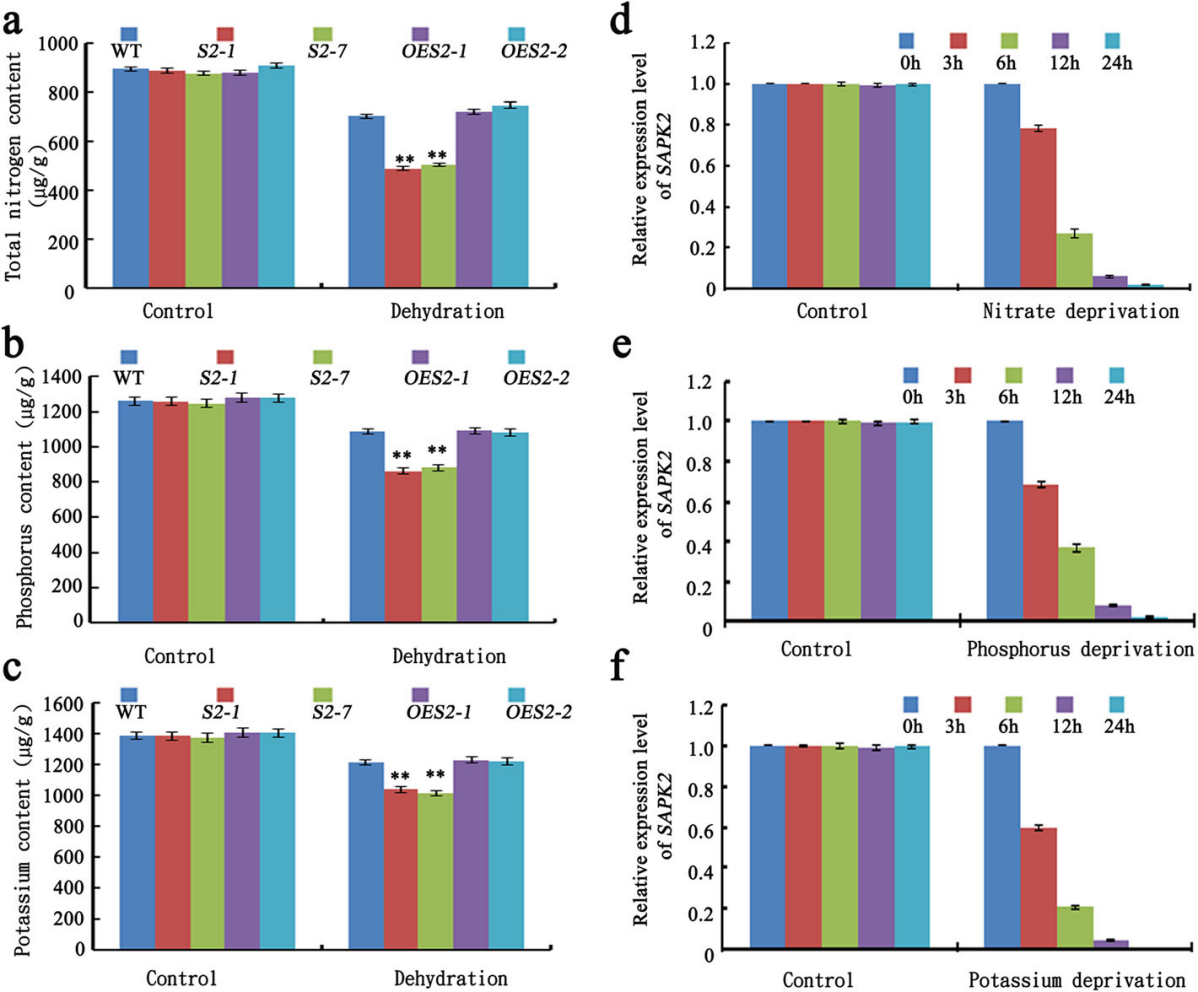
*SAPK2* influenced Nitrate, phosphorus, potassium content in rice grains under reproductive-stage drought. **a-c** Comparison of nutrient content including total nitrogen (**a**), phosphorus (**b**), potassium (**c**) among WT, *sapk2* mutant lines and *OE* lines in rice seeds under reproductive-stage drought. **d-f** The expression analysis of *SAPK2* under N (**d**), P (**e**), and K (**f**) deprivation. Transcript accumulation was assessed by qRT-PCR. Data are shown as means ± SD (*n* = 20) from three replicates. A student’s *t*-test was used to generate *P* values; “**” indicate significance at *P* < 0.01

Umezawa et al. (2004) reported that the expression of *SnRK2.8*, which is a homolog of rice *SAPK2*, is down-regulated by potassium deprivation. This down-regulation is associated with a substantial decrease in the growth of *A. thaliana* under nutrient-deprived conditions. As mentioned earlier, SAPK2 influences rice panicle and grain sizes. To clarify the mechanisms by which SAPK2 influences panicle and grain sizes in rice under RDS, we measured total nitrogen, nitrate, phosphorus, and potassium contents of seeds. Under RDS conditions, the seeds of the *sapk2* mutant lines had lower total nitrogen, nitrate, phosphorus, and potassium content than WT, with the biggest difference observed for the total nitrogen (Fig. 3a–c, Additional file 2: Figure S1). However, the total nitrogen, nitrate, phosphorus, and potassium content were relatively consistent between the *OE* and WT plants (Fig. 3a–c).

Next, we investigated the *SAPK2* expression profiles under control conditions (i.e., sufficient nutrients) and nutrient-deficient conditions [i.e., lacking K (−K), N (−N), and P (−P)]. The qRT-PCR analyses revealed that the *SAPK2* transcript levels in the roots decreased in the absence of N, P, and K (Fig. 3d–f).

These findings confirmed that in rice, the seed nitrate, phosphorus, and potassium contents are largely affected by SAPK2. Therefore, we hypothesized that SAPK2 influences panicle and grain sizes by modulating metabolic processes of N, P, and K.

### SAPK2 Affects Seedling Growth and Root Development in Response to N and K Deprivation

To further validate our hypothesis, we investigated the effects of knocking out and overexpressing *SAPK2* on rice seedling growth and development in hydroponic cultures under different nutrient-deprived (−K, −N, and − P) conditions.

**Fig. 4:**
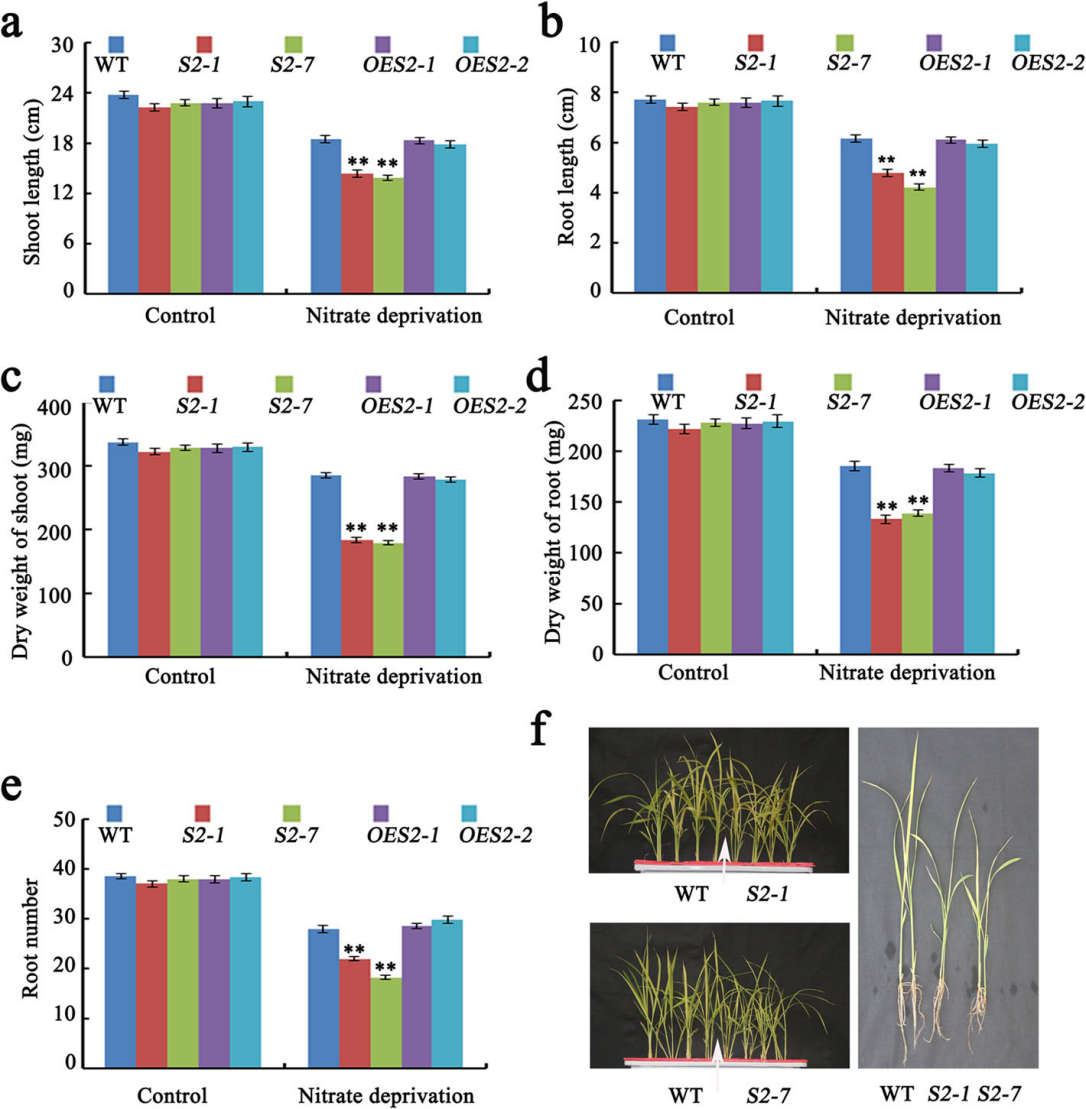
*SAPK2* affects seedling growth and root development under N deprivation. **a-e** Statistical analysis of shoot length (**a**), root length (**b**), dry weight of shoot (**c**), dry weight of root (**d**) and root number (**e**) among WT, *sapk2* mutant lines and *OE* lines under N-deprived conditions. **f** Phenotypic analysis of seedlings at 31 DAG among WT, *sapk2* mutant lines and *OE* lines under N-deprived conditions. Data in **a-e** are shown as means ± SD (*n* = 20) from three replicates. A student’s *t*-test was used to generate *P* values; “**” indicate significance at *P* < 0.01

The *sapk2* mutant seedlings under N-deprived conditions produced weaker culms than WT, whereas the *OE* lines were phenotypically similar to WT (Fig. 4a, f; In Fig. 4f, the *OE* phenotype is not presented). A previous study proved that the root morphology influences plant interactions with soil nitrates, making it important for N absorption (Hachiya and Sakakibara 2016). Accordingly, we examined the root development of the *SAPK2* transgenic lines. In the *sapk2* mutant lines, root growth was inhibited, resulting in roots than WT seedlings (Fig. 4b, f). In contrast, the root phenotypes of the *OE* and WT plants were similar (Fig. 4b, f; In Fig. 4f, *OE* phenotype is not presented). The root and shoot dry weights of the *sapk2* mutant lines were significantly lower than WT, but there were no significant differences in the *OE* lines (Fig. 4c, d). Similarly, compared with WT, the *sapk2* mutant lines had fewer roots, whereas there were no significant differences in the *OE* lines (Fig. 4e).

The effects of the K-deprived conditions were similar to those of the N-deprived conditions. For example, the *sapk2* mutant seedlings produced weaker culms and had lower root and shoot dry weights than WT (Additional file 3: Figure S2a–f). In contrast, the exposure to P-deprived conditions did not result in any significant differences in the seedling growth and root development of the WT, *OE* and *sapk2* seedlings (Additional file 4: Figure S3a–f).

These findings suggested that SAPK2 can significantly influence rice seedling growth and root development in hydroponic cultures under N- and K-deprived conditions.

### SAPK2 Influences the NO_3_^−^ Influx Rate and Nitrate Concentration under Drought RDS

**Fig. 5:**
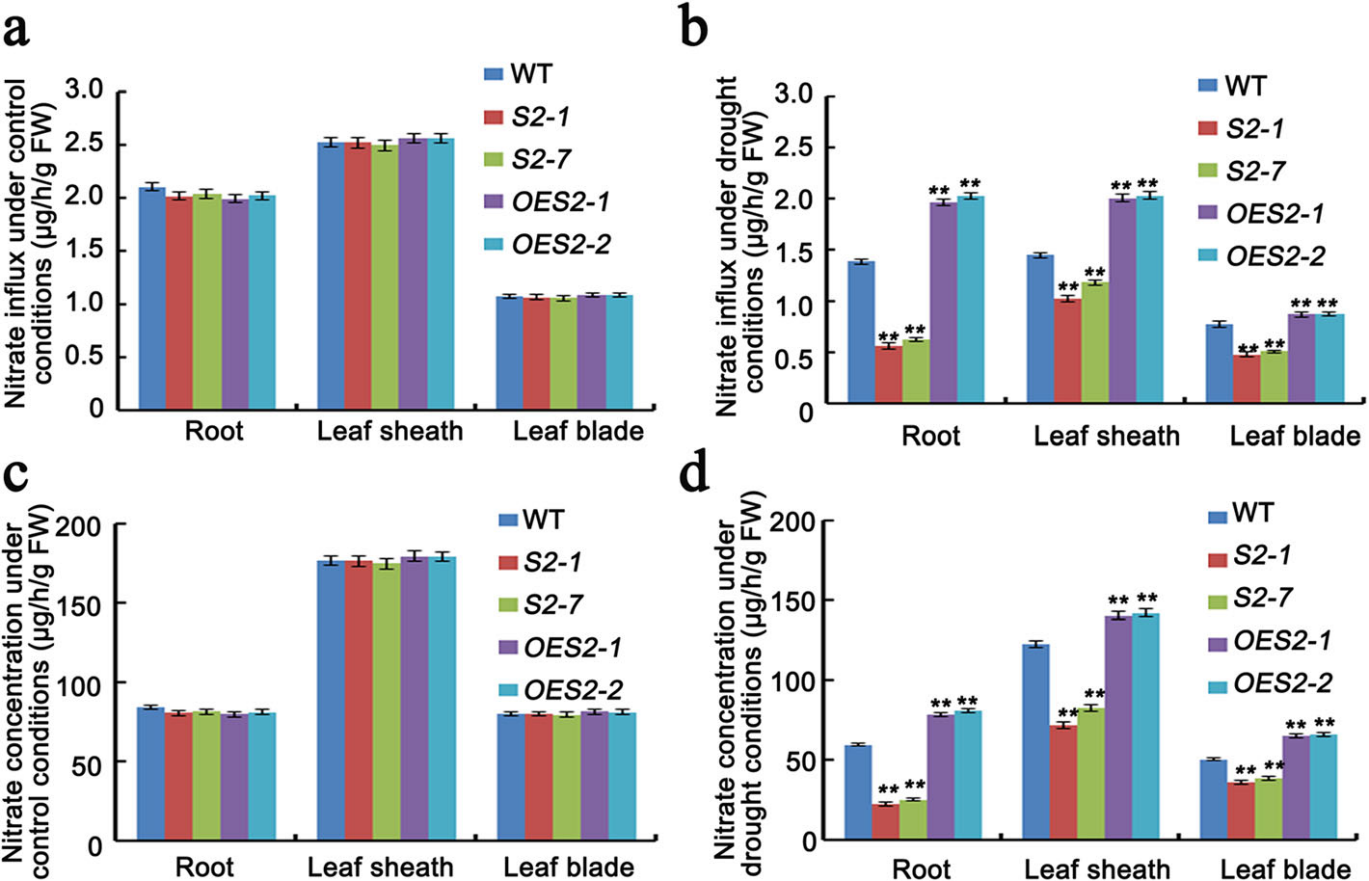
*SAPK2* influenced the rate of NO_3_^−^ influx rate and nitrate concentration. **a-b** Analysis of NO_3_^−^ influx rate among WT, *sapk2* mutant lines and *OE* lines under control conditions (**a**) and drought stress conditions (**b**). **c-d** Analysis of nitrate concentration among WT, *sapk2* mutant lines and *OE* lines under control conditions (**c**) and drought stress conditions (**d**). Data are shown as means ± SD (*n* = 20) from three replicates. A student’s *t*-test was used to generate *P* values; “**” indicate significance at *P* < 0.01

To explore the potential mechanism underlying the effects of SAPK2 on rice seedling growth and root development under N-deprived condition, we investigated the NO_3_^−^ influx rate and nitrate concentration of the WT, *OE*, and *sapk2* plants under control and drought conditions. Under control conditions, there were no significant differences among the WT, *OE*, and *sapk2* plants (Fig. 5a, c). However, in response to drought stress, the NO_3_^−^ influx rate and nitrate concentration significantly decreased in the WT, *OE*, and *sapk2* plants (Fig. 5a–d). Additionally, the rate of NO_3_^−^ influx into the roots was lower for the *sapk2* mutant lines than for WT (Fig. 5b), suggesting that knocking out of *SAPK2* weakens the nitrate uptake by the roots. Regarding the *sapk2* mutant lines, we also detected a lower rate of NO_3_^−^ influx into the leaf sheath and leaf blade, implying that SAPK2 promotes the translocation of NO_3_^−^ from the roots to the leaf sheath (Fig. 5b). Moreover, the root, leaf sheath, and leaf blade nitrate concentrations were consistent with the NO_3_^−^ influx rates in the different lines (Fig. 5d). These results demonstrated that SAPK2 enhances nitrate influx and increases the nitrate concentration by promoting the translocation of nitrate from the roots to the leaf sheath.

Nitrogen use efficiency is an important trait for the development of sustainable agricultural production (Xu et al. 2012). Plants have diverse transporters facilitating N uptake and internal distribution (Rentsch et al. 2007). In higher plants, members of the NPF family (previously called the PTR/NRT1 family) can take up and translocate nitrate or small peptides. Of the rice NPF family members, only a few have been studied. For example, *OsNPF7.2*, which encodes a positive regulator of nitrate influx and concentration, helps control the allocation of nitrate between the roots and shoots (Wang et al. 2018). In rice, the peptide transporter OsNPF7.3 (OsPTR6) mediates the transport of organic N from the leaves to the grains and increases the grain yield (Fang et al. 2017). Additionally, *OsNPF6.5* (*OsNRT1.1B*) is predominantly expressed in the root hairs, epidermis, and stellar cells adjacent to the xylem in roots. The *osnrt1.1b* mutant is reportedly defective in both nitrate uptake and root-to-shoot nitrate transport, suggesting that OsNRT1.1B is involved in nitrate uptake and transport (Hu et al. 2015). Down-regulating *OsNRT2.3a* expression impairs the loading of nitrate into the xylem and inhibits plant growth under low-nitrate conditions, implying OsNRT2.3a contributes to the long-distance transport of nitrate from the roots to the shoots (Tang et al. 2012). The silencing of *OsNPF2.4* diminishes the low-affinity nitrate acquisition by roots, disrupts the K-coupled root-to-shoot nitrate transport, and inhibits the redistribution of nitrate from old leaves to N-starved roots or young leaves (Xia et al. 2015).

**Fig. 6:**
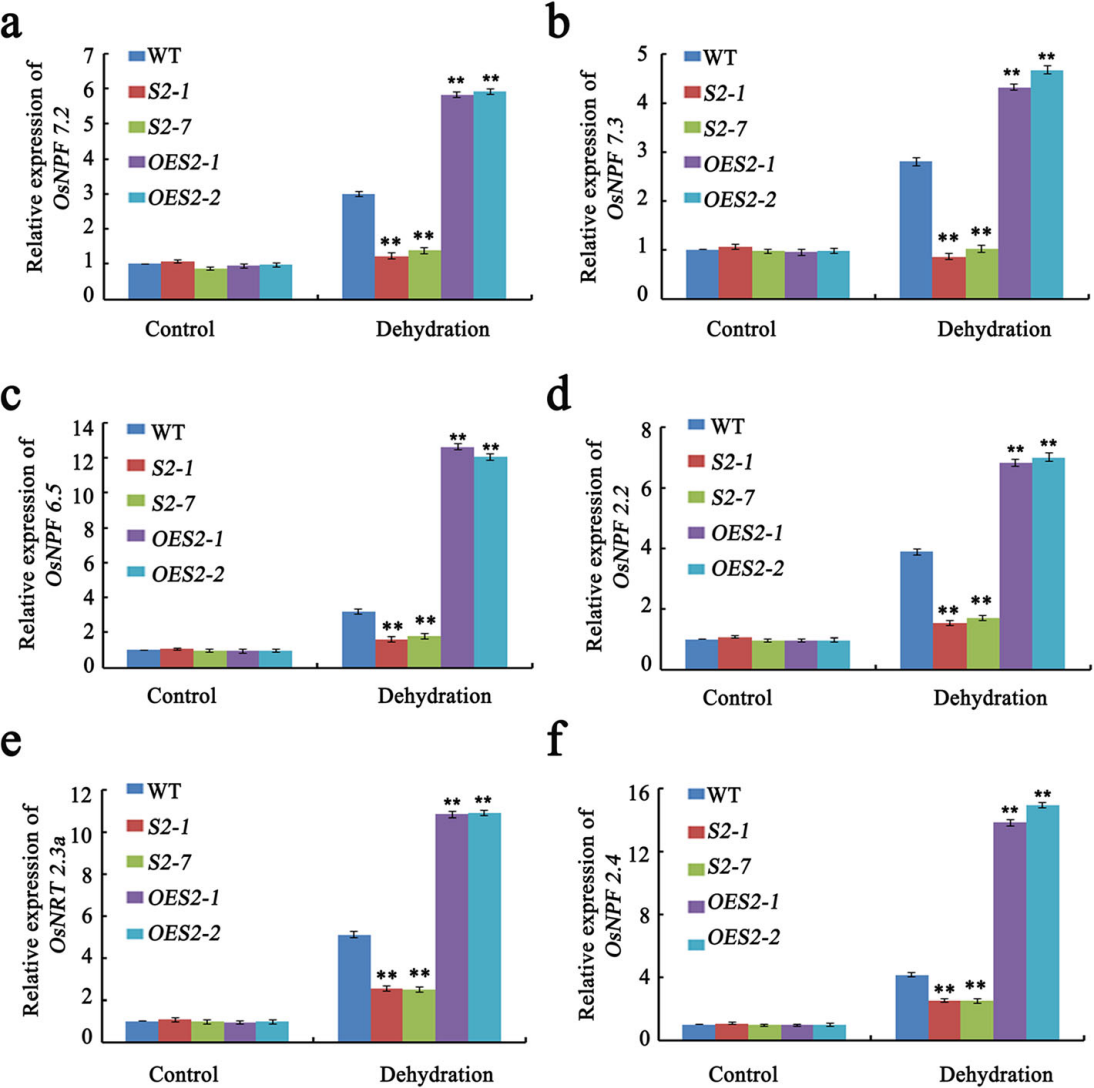
Expression of genes involved in absorbing, transporting and assimilation of nitrate among WT, OE lines and *sapk2* mutant lines. **a-f** Relative expression analysis of *OsNPF7.2* (**a**), *OsNPF7.3* (**b**)*, OsNPF5.6***(c)***, OsNPF2.2* (**d**), *OsNRT2.3a* (**e**) and *OsNPF2.4* (**f**) among WT, *sapk2* mutant lines and *OE* lines control conditions and drought stress conditions. Data are shown as means ± SD (*n* = 20) from three replicates. A student’s *t*-test was used to generate *P* values; “**” indicate significance at *P* < 0.01

To further validate our hypothesis that SAPK2 influences panicle and grain sizes by modulating N metabolic processes, we determined the expression levels of genes crucial for the absorption, transport, and assimilation of nitrate among the WT, *OE*, and *sapk2* plants cultured under control and drought stress condition (PEG). The *OsNPF7.2*, *OsNPF7.3*, *OsNPF5.6*, *OsNPF2.2*, *OsNRT2.3a*, and *OsNPF2.4* expression levels were significantly lower in the *sapk2* mutant lines than in WT under drought stress condition (Fig. 6a–f). However, the opposite expression patterns were detected for the *OE* lines (Fig. 6a–f). These results implied that SAPK2 promotes nitrate uptake and assimilation by regulating nitrate-related transporters.

## Discussion

### Mutations to *SAPK2* Decrease Grain Yield

Drought is one of the most important environmental stresses affecting the productivity of most field crops (Gray and Brady 2016). Elucidating the complex mechanisms underlying the drought resistance of crops will accelerate the development of new varieties with enhanced drought resistance. Improving the grain yield is the primary aim for most rice breeders. The rice yield potential is determined by the biomass and harvest index.

To identify additional rice grain yield-related genes affected by drought stress, we functionally characterized *SAPK2* by examining *sapk2* mutant lines exposed to drought stress. Under RDS condition, the *sapk2* mutant plants produced fewer effective tillers and grains per plant than the WT and *OE* plants. Rice grain yield is determined by the following three traits: number of panicles, number of grains per panicle, and grain weight (Xing and Zhang 2010). Consequently, we were interested in whether *SAPK2* can be used to improve rice yields under RDS conditions. We also investigated the SAPK2 roles associated with panicle and grain development in response to RDS conditions. Specifically, the panicle size, number of grains per panicle, grain size, setting rate, 1000-grain weight and grain yield per plant were significantly lower for the *sapk2* mutant lines than WT under RDS (Fig. 2a–g). These results indicated that SAPK2 increases rice yield under RDS by influencing panicle and grain size.

### SAPK2 Affects Seedling Growth and Root Development in Response to N and K Deprivation

A previous study revealed that SRK2C/SnRK2.8 may mediate the phosphorylation of enzymes involved in metabolic processes (Umezawa et al. 2004). To investigate whether the decrease in the grain yield of the *sapk2* mutant lines under RDS is related to nutrient metabolism, we measured the seed total nitrogen, nitrate, phosphorus, and potassium content. The data indicated the *sapk2* mutant seeds had lower total nitrogen, nitrate, phosphorus, and potassium content than that in WT and *OE* lines seeds, especially for total nitrogen content (Fig. 3a–c and Additional file 2: Figure S1).

The roots, which are responsible for the uptake of water and nutrients from the soil, are vital for plant growth, development, and fitness. Additionally, ABA controls several key steps associated with lateral root initiation as well as meristem activation and elongation (De Smet et al. 2003; Ding and De Smet 2013). Root development is directly affected by environmental factors. Furthermore, the plant root system architecture is plastic and dynamic; enabling plants to respond to environmental changes, which then promotes root growth and development to avoid water deficit stress in the early stages of drought stress. Nitrogen availability is a major determinant of plant growth and crop productivity (Hachiya and Sakakibara 2016; Li et al. 2017). Plants use inorganic forms in natural soils, including nitrates, nitrites, and ammonium, and nitrate is the major form of N in most aerated soils. Of these available N sources, researchers have mainly focused on nitrate and ammonium because these are often present in natural and cropland soils at much higher levels than the other N sources (Miller and Cramer 2004).

To further validate the relationship between SAPK2 and N metabolism, we investigated the effects of knocking out and overexpressing *SAPK2* on rice seedling growth and development in hydroponic cultures under N-deprived conditions. The *sapk2* mutant seedlings under N-deprived conditions had weaker culms than WT, with inhibited root growth, significantly lower root and shoot dry weights, and fewer roots (Fig. 4a–f). However, we did not find significant difference between WT and *OE* plants (Fig. 4a–e).

Earlier investigations confirmed the importance of K for enzyme activities and ionic homeostasis in plants (Shabala and Pottosin 2014; Ahmad and Maathuis 2014). Additionally, we previously determined that SAPK2 regulates the expression of genes related to Na^+^ and K^+^ homeostasis, including *OsSOS1*, *OsNHX1*, *OsHKT1;1*, and *OsHKT1;5* (lou et al. 2018). In this study, the *sapk2* mutant seedlings deprived of K produced weaker culms and had lower root and shoot dry weights than WT and *OE* plants (Additional file 3: Figure S2).

Furthermore, we also investigated *SAPK2* effects on rice seedling growth and development in hydroponic cultures under P-deprived conditions and found there was no significant difference between *sapk2* mutant, WT and *OE* plants (Additional file 4: Figure S3).

Therefore, above results presented here confirm that SAPK2 influences panicle and grain sizes via its effect on N and P metabolic processes and is positive regulator in response to N and K deprived conditions.

However, the SAPK2 expression was down-regulated in response to N, P, and K deprivation in the roots (Fig. 3d–f). We think the reason for this result is related to how SAPK2 works. SAPK2 is a member of SnRK2 subclass II in rice. In the absence of ABA or osmotic stress, SAPK2is inhibited by ABA receptors OsPYL/RCAR5 and induces complex formation with PP2C30 through dephosphorylating. Under ABA or osmotic stress, ABA accumulates and binds to its receptors OsPYL/RCAR5, subsequently inhibit PP2C30 activity, resulting in the release of SAPK2 from inhibition. Activated SAPK2 phosphorylate downstream effectors to mediate stress responses (Kim et al. 2012). Current models suggest that ABA, which is induced by drought stress, inhibits the activity of OsPP2C49 to release the kinase activity of SAPK2 for further activation of OsbZIP23 through phosphorylation. OsbZIP23 could directly bind to the promoters of OsPP2C49 and positively regulates the expression of OsPP2C49, which in turn negatively regulates the ABA signaling (Zong et al. 2016). This regulatory mechanism of SAPK2 mainly occurs at the protein level, but does not change much at *SAPK2* transcription level. However, there may be a negative regulatory mechanism at SAPK2 transcription level after this response, resulting in a decrease in *SAPK2* transcription level.

### SAPK2 Influences the NO_3_^−^ Influx Rate and Nitrate Concentration

Nitrogen uptake, assimilation, and recycling in plant roots reportedly determine plant development and productivity (Yamaya and Kusano 2014). Plant growth under natural conditions is often limited by N availability. Therefore, plants have developed transport and signaling mechanisms for their specific N sources (Kiba and Krapp 2016). To further validate the relationship between SAPK2 and nitrogen absorption and utilization by investigating the effects of silencing and overexpressing *SAPK2* on rice. Under drought stress conditions, in the *sapk2* mutant lines, we detected a lower rate of NO_3_^−^ influx into roots and also into the leaf sheath and leaf blade (Fig. 5b). Besides, the detected nitrate concentration was consistent with the rate of NO_3_^−^ influx in different lines (Fig. 5d). These results demonstrated that *SAPK2* enhanced nitrate influx and concentration by promoting nitrate uptake by roots and translocation of nitrate from roots to leaf sheath.

Although there are more than 80 NRT1/PTR, 4 NRT2, and 2 NAR2 members in rice, only a few NRT1/PTR family members have been characterized (Araki and Hasegawa 2006; Cai et al. 2008; Feng et al. 2011). Additionally, only a few nitrate transporters (OsNPF7.2, OsNPF7.3, OsNPF6.5, OsNPF2.2, OsNRT2.3a, and OsNPF2.4) have been characterized in rice (Wang et al. 2018; Fang et al. 2017; Hu et al. 2015; Li et al. 2015; Tang et al. 2012; Xia et al. 2015).

In this study, we analyzed the expression of several nitrate transporter genes (*OsNPF7.2*, *OsNPF7.3*, *OsNPF5.6*, *OsNPF2.2*, *OsNRT2.3a*, and *OsNPF2.4*). Under drought conditions, the expression levels of these six genes were significantly lower in the *sapk2* mutant lines than in the WT plants in response to drought stress (Fig. 6a–f). Furthermore, OsNPF6.5 regulates the number of rice tillers and promotes grain production (Hu et al. 2015). The overexpression of *OsNPF7.2* and *OsNPF7.3* increases the production of tillers, panicles per plant, and filled grains per panicle, while also increasing the grain N content (Wang et al. 2018; Fang et al. 2017). The *osnpf2.2* (*OsPTR2*) mutants are defective in long-distance nitrate transport, with repressed nitrate unloading from the xylem, resulting in plant growth retardation and abnormal grain filling (Li et al. 2015). Our study revealed that the *sapk2* mutant lines produced significantly fewer tillers than the WT plants (Fig. 1d). Moreover, the *sapk2* mutant seeds had a lower total nitrogen content than WT seeds under RDS condition (Fig. 3a).

On the basis of our results, we conclude that under drought stress, SAPK2 promotes nitrate uptake and assimilation and influences the number of tillers and the number of grains per panicle by regulating nitrate-related transporters.

## Conclusions

In this study, we examined knock-out mutant lines and *SAPK2*-overexpressing lines. We found that the *sapk2* mutant showed lower grains yield and lower nitrogen, phosphorus, and potassium contents of rice grains than the WT under RDS. Moreover, the *sapk2* mutant exhibited weaker seedling growth and root development in hydroponic cultures lacking N and K. The NO_3_^−^ influx rate and nitrate concentration analysis indicated that SAPK2 promotes nitrate uptake and assimilation by regulating nitrate-related transporters. These results suggest that *SAPK2* could enhance grain production by regulating nitrogen utilization efficiency under RDS. Our work provided insights to breeding drought tolerant rice with high nutrient uptake.

## Supplementary information

**Additional file 1: Table S1.** Primers used in this study.

**Additional file 2: Figure S1.** The comparison of nutrient content including nitrate among WT, *sapk2* mutant lines and *OE* lines in rice seeds under RDS.

**Additional file 3: Figure S2.***SAPK2* affects seedling growth and root development under K deprivation. **a-e** Statistical analysis of shoot length **(a)**, root length **(b)**, dry weight of shoot **(c)**, dry weight of root **(d)** and root number **(e)** among WT, *sapk2* mutant lines and *OE* lines under K-deprived conditions. **f** Phenotypic analysis of seedlings at 31 DAG among WT, *sapk2* mutant lines and *OE* lines under K-deprived conditions. Data in **a-e** are shown as means ± SD (*n* = 20) from three replicates. A student’s *t*-test was used to generate *P* values; “**” indicate significance at *P* < 0.01.

**Additional file 4: Figure S3.***SAPK2* affects seedling growth and root development under P deprivation. **a-e** Statistical analysis of shoot length **(a)**, root length **(b)**, dry weight of shoot **(c)**, dry weight of root **(d)** and root number **(e)** among WT, *sapk2* mutant lines and *OE* lines under P-deprived conditions. **f** Phenotypic analysis of seedlings at 31 DAG among WT, *sapk2* mutant lines and *OE* lines under P-deprived conditions. Data in **a-e** are shown as means ± SD (*n* = 20) from three replicates. A student’s *t*-test was used to generate *P* values; “**” indicate significance at *P* < 0.01.

## Data Availability

All data generated or analyzed during this study are included in this published article and its additional files.

## Abbreviations

SAPK2: osmotic stress/ABA–activated protein kinase 2
*S2–1*: *sapk2–1*
*S2–7*: *sapk2–7*
*OE*: Overexpression
WT: Wild type
RDS: Reproductive stage drought stress
ROS: Reactive oxygen species
JA: Jasmonic acid
ABA: Abscisic acid
SA: Salicylic acid
ET: Ethylene
Gas: Gibberellins
CKs: Cytokinins
RS: Reproductive-stage drought
qRT-PCR: Quantitative Reverse Transcription Polymerase Chain Reaction
DAG: Days after germination
