# Supplementary material for: SAPK2 contributes to rice yield by modulating nitrogen metabolic processes under reproductive stage drought stress
Source: Rice (N Y). 2020 Jun 8;13:35. doi: 10.1186/s12284-020-00395-3 (PMC7280414; doi:10.1186/s12284-020-00395-3)
Supplement: Supplementary file 1 — Additional file 1: Table S1. Primers used in this study. [file 12284_2020_395_MOESM1_ESM.docx]

**Additional file 1. Primers and oligos used in this study**

| Name | Primer sequence (5’-3’) |
| --- | --- |
| **Plasmid Constrcutions and Mutation Detection** | |
| U3-*SAPK2*-F | GGCATAGTTATGGAATATGCTGC |
| U3-*SAPK2-*R | ATCAATACCTTATACGACGCAAA |
| *SAPK2*-u3-F | GGAAATGAAAGTGACCCAGCAG |
| *SAPK2*-u3-R | TGCTCCCATCCAAGAGAGTATTTT |
| Promoter-*SAPK2*-F | ATAGTCGACAAGATCCTGGGATCAAAGAAAGCTTCG |
| Promoter-*SAPK2*-R | ATAGGATCCCCCCACCTCCCACGACAACTCC |
| P30-*SAPK2*-F | ATAGGATCCATGGAGAGGTACGAGGTGATCAAGGACAT |
| P30-*SAPK2*-R | ATATCTAGACAATGCGCACACGAAGTCGC |
| P1301-*SAPK2*-F | ATAGGATCCATGGAGAGGTACGAGGTGATCAAGGACAT |
| P1301*-SAPK2*-R | ATAGGTACCTCACAATGCGCACACGAAGTCGC |
| ***qRT-PCR*** | |
| OsActin-RT-F | AGCTGCGGGTATCCATGAGA |
| OsActin-RT-R | GCAATGCCAGGGAACATAGTG |
| RT-*OsNPF7.2*-F | TGCAAGTGCCACTCCTCAAGG |
| RT-*OsNPF7.2*-R | AGGACGGTCTCCAGGTACACCACC |
| RT-*OsNPF7.3*-F | ACTCCAGGACGACGTCAGCCT |
| RT-*OsNPF7.3*-R | ACCAGGTTCGTGGCAATGCCGT |
| RT-*OsNPF6.5*-F | TGGCGATGGTGTTGCCGGAGA |
| RT-*OsNPF6.5*-R | AACAGCTCCGCCCCGAGGATCA |
| RT-*OsNPF2.2*-F | ACGGCGAACCTGCTGGTGTACC |
| RT-*OsNPF2.2*-R | TGAGGGTGAGCACAAGCATGCCGA |
| RT-*OsNRT2.3a*-F | GCGAGAAGGGTTTCAACGCGGCCA |
| RT-*OsNRT2.3a*-R | ACCGCGACCTTATTGTCCGTGGCA |
| RT-*OsNPF2.4*-F | ACCAGCACTGGATGTCGCGGATCT |
| RT-*OsNPF2.4*-R | ACGAGCATGGCGCACGGGAGTA |
