# Supplementary figures and images for: SAPK2 contributes to rice yield by modulating nitrogen metabolic processes under reproductive stage drought stress
Source: Rice (N Y). 2020 Jun 8;13:35. doi: 10.1186/s12284-020-00395-3 (PMC7280414; doi:10.1186/s12284-020-00395-3)

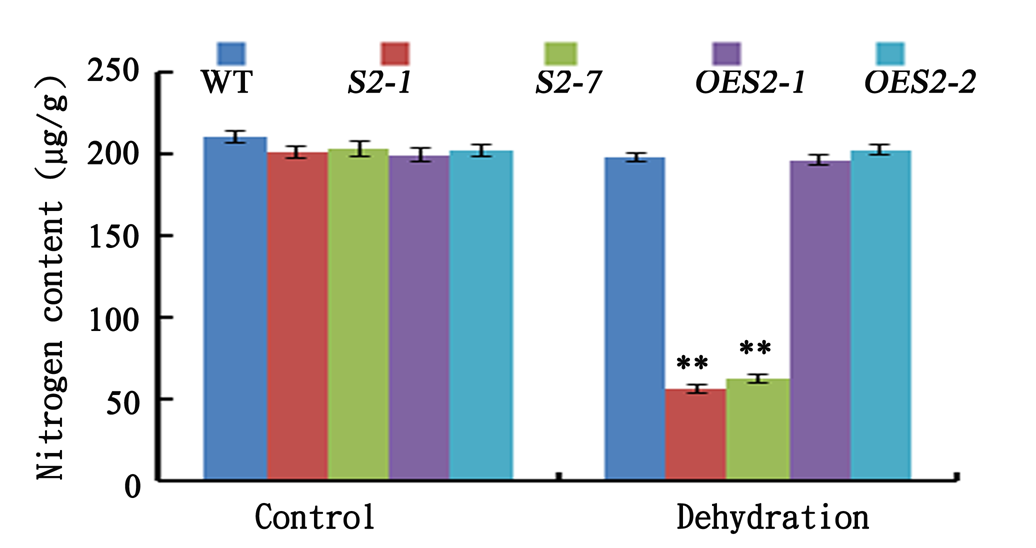

Supplement: Supplementary file 2 — Additional file 2: Figure S1. The comparison of nutrient content including nitrate among WT, sapk2 mutant lines and OE lines in rice seeds under RDS. [file 12284_2020_395_MOESM2_ESM.tif]

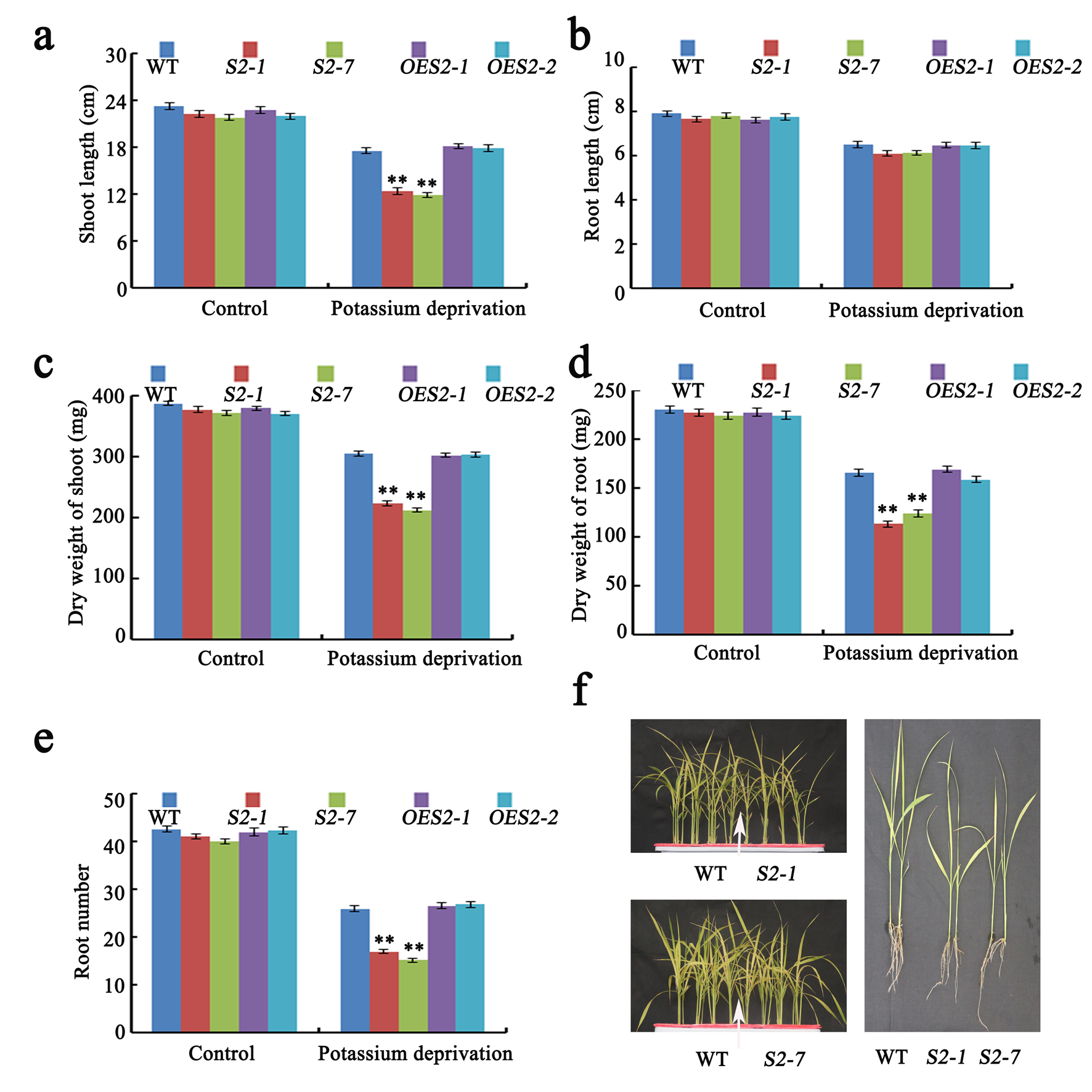

Supplement: Supplementary file 3 — Additional file 3: Figure S2.SAPK2 affects seedling growth and root development under K deprivation. a-e Statistical analysis of shoot length (a), root length (b), dry weight of shoot (c), dry weight of root (d) and root number (e) among WT, sapk2 mutant lines and OE lines under K-deprived conditions. f Phenotypic analysis of seedlings at 31 DAG among WT, sapk2 mutant lines and OE lines under K-deprived conditions. Data in a-e are shown as means ± SD (n = 20) from three replicates. A student’s t-test was used to generate P values; “**” indicate significance at P < 0.01. [file 12284_2020_395_MOESM3_ESM.tif]

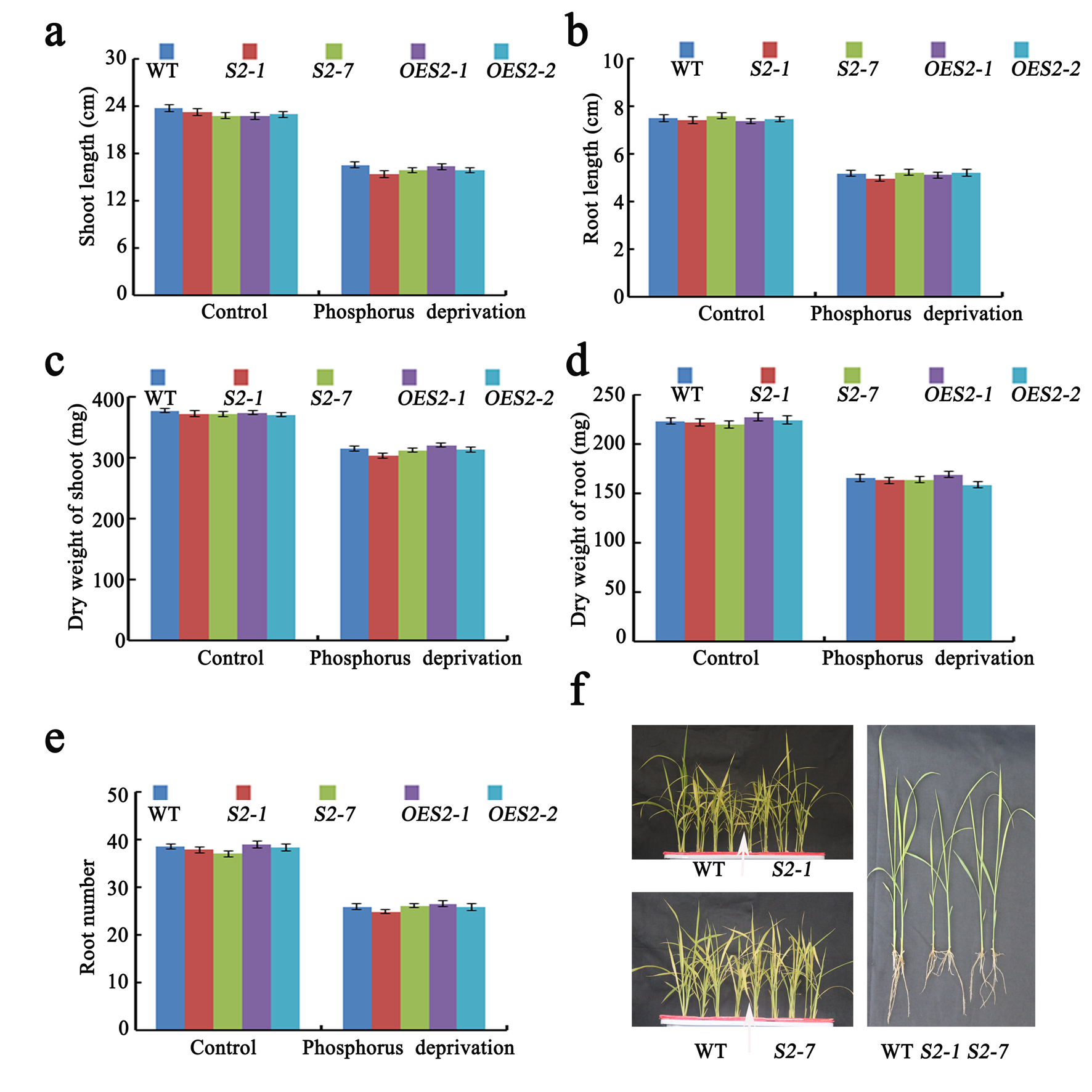

Supplement: Supplementary file 4 — Additional file 4: Figure S3.SAPK2 affects seedling growth and root development under P deprivation. a-e Statistical analysis of shoot length (a), root length (b), dry weight of shoot (c), dry weight of root (d) and root number (e) among WT, sapk2 mutant lines and OE lines under P-deprived conditions. f Phenotypic analysis of seedlings at 31 DAG among WT, sapk2 mutant lines and OE lines under P-deprived conditions. Data in a-e are shown as means ± SD (n = 20) from three replicates. A student’s t-test was used to generate P values; “**” indicate significance at P < 0.01. [file 12284_2020_395_MOESM4_ESM.tif]
